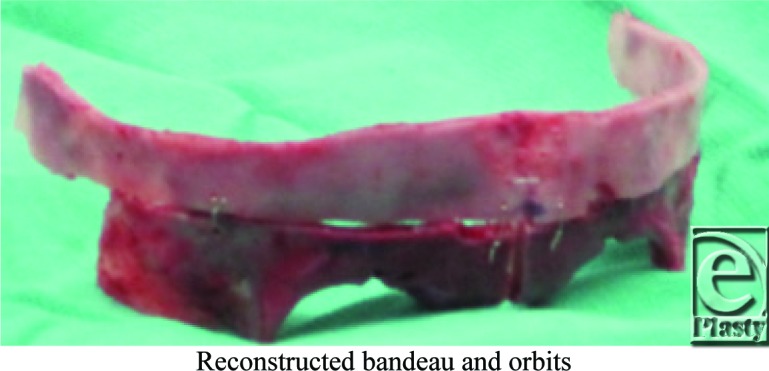# Craniosynostosis in a 22-Month-Old

**Published:** 2013-01-25

**Authors:** Ian C. Hoppe, Frank S. Ciminello

**Affiliations:** Division of Plastic Surgery, New Jersey Medical School, University of Medicine and Dentistry, Newark, NJ

**Figure:**
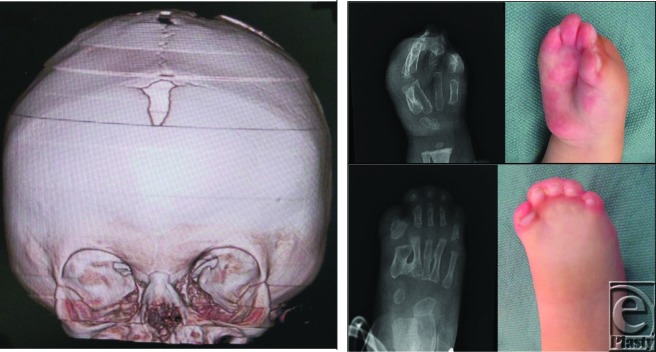


## DESCRIPTION

A 22-month-old child presents with craniosynostosis and the pictured limb deformities.

## QUESTIONS

1. **What is the most likely diagnosis and what is its etiology?**
  **Describe repair of the cranial deformity.**
2. **What other procedures may be necessary for this child?**

## DISCUSSION

Syndromic craniosynostosis such as Crouzon, Apert, Pfeiffer, Saethre-Chotzen, and Carpenter syndromes are represented by a common set of calvarial, midface, and hand deformities. The syndactyly in this case makes the diagnosis of Apert syndrome (acrocephalosyndactyly) most likely. Other physical features include craniosynostosis, hypertelorism, and midface hypoplasia. Defects in the fibroblast growth receptor 2 gene on chromosome 10 and the fibroblast growth receptor 3 gene on chromosome 4 have been implicated. It is inherited in an autosomal dominant fashion, but most cases involve sporadic mutations. Approximately 50% of children born with Apert syndrome experience mild to moderate mental retardation.

Surgical repair of the cranial deformities involves cranial vault expansion and fronto-orbital advancement to reduce the exophthalmos as well as reduce intracranial pressure. This procedure is performed prior to 1 year of age to maximize the reparative abilities of the calvarium and to reduce the reported incidence of cognitive delays.

The limb abnormalities warrant extensive reconstruction and present the surgeon with one of the most complex congenital limb deformities. Return of rudimentary function can be expected following a multistage reconstruction. The most important procedure is the opening of the first and fourth web spaces.

## Figures and Tables

**Figure:**